# Assessment of Albumin Therapy and Paracentesis Interval in Cirrhotic Patients With Recurrent Ascites: A Prospective Cohort Study

**DOI:** 10.7759/cureus.86016

**Published:** 2025-06-14

**Authors:** Muhammad Abdullah Khan, Hafiz Muhammad Faizan Mughal, Shehwar Ahmed, M Khaliq, Abdul Ghafoor

**Affiliations:** 1 General Medicine, King's Mill Hospital, Sutton-In-Ashfield, GBR; 2 Internal Medicine, Khawaja Muhammad Safdar Medical College, Sialkot, PAK; 3 Medicine, Sargodha Medical College, Sargodha, PAK; 4 Pathology, University of Health Sciences Lahore, Lahore, PAK; 5 Medicine, Bolan University of Medical and Health Sciences, Quetta, PAK

**Keywords:** albumin therapy, cirrhosis, fluid management, paracentesis, picd, recurrent ascites, renal dysfunction

## Abstract

Background and aim: Many people with liver cirrhosis develop recurrent ascites that requires repeated treatments of large-volume paracentesis (LVP). This study aimed to determine whether intravenous albumin treatment extends the interval between paracentesis procedures, while also preventing paracentesis-induced circulatory dysfunction (PICD) and renal dysfunction among cirrhotic patients who experience recurrent ascites.

Methods: This prospective cohort study was carried out at a tertiary care hospital in Pakistan from April 2023 to August 2023, including 120 patients undergoing LVP treatment for cirrhosis were divided into two equal groups. OpenEpi version 3.0.0 (released 2013, developed by Andrew G. Dean, Kevin M. Sullivan, and Daniel G. Soe, Atlanta, GA, USA) was used for calculation. The 60 patients in Group A received albumin, while 60 in Group B received only saline. Over four months, outcomes were evaluated. The statistical analysis was performed using IBM SPSS Statistics for Windows, Version 26 (Released 2019; IBM Corp., Armonk, New York, United States).

Results: Group A experienced a prolonged time interval between paracentesis treatments (18.2 vs. 11.1 days, p < 0.001) and a reduced occurrence of PICD (7 (11.7%) vs. 23 (38.3%), p = 0.001) and renal dysfunction (6 (10.0%) vs. 18 (30.0%), p = 0.008). The patients in Group A experienced reduced incidents of hospitalizations, together with emergency procedures. This highlighted the greater efficacy of albumin than saline.

Conclusion: Using albumin in therapy decreased the risks of recurring fluid buildup in cirrhotic patients, as it helped in prolonging the time between repeated fluid removal procedures. However, due to a single-center approach, the generalizability of the results was limited. Nonetheless, albumin administration at the standardized intervals should become part of the therapeutic regimen because it ensures patients' safety and reduces the workload on medical staff.

## Introduction

Liver cirrhosis is a worldwide public health issue that is often complicated by the development of ascites, which is a sign of decompensated cirrhosis [1]. Recurrent ascites not only indicates disease progression but also significantly reduces the quality of life, increases healthcare utilization, and is also a significant predictor of mortality [2]. Among the standardized management approaches, large-volume paracentesis (LVP) is usually necessary for relief in patients with refractory or recurrent ascites. Nevertheless, repeated LVP is not entirely without risks, and one notable complication is paracentesis-induced circulatory dysfunction (PICD), which may trigger renal impairment and predispose to morbidity [3].

To prevent these risks, intravenous albumin is commonly used as a post-paracentesis therapy as it functions as a plasma expander. Albumin is thought to maintain circulatory stability, decrease PICD incidence, and potentially promote the perfusion of kidneys [4,5]. Despite these theoretical advantages and current clinical guidelines that recommend its use, the precise frequency and long-term effectiveness of albumin therapy for the reduction of complications related to paracentesis and hospital readmissions continued to be the subject of ongoing research [6].

Due to the high cost of albumin, its usage is limited in areas where resources are inadequate, i.e., healthcare facilities in developing countries [7,8]. This economic burden made it essential to determine the actual clinical benefits of albumin in prolonging the paracentesis-free intervals and improving patient outcomes. Moreover, an awareness of comparative effects of albumin versus more affordable options, such as saline, could provide evidence-based clinical protocols that could balance the therapeutic efficacy with the practical feasibility.

A study had been designed to investigate the effects of intravenous albumin treatment on recurrent ascites in liver cirrhosis patients. This study was conducted to compare the effects of albumin versus saline, focusing on the key outcomes like the interval between paracenteses, the incidence of PICD, preservation of renal function, and hospitalization rates. The study sought to determine the albumin's therapeutic benefits for cirrhotic patients with ascites to suggest optimal treatment approaches.

## Materials and methods

This prospective cohort study was conducted at the Federal Postgraduate Medical Institute, Lahore, in Pakistan during the period from April 2023 to August 2023. The hospital received approval from the Institutional Review Board of the Federal Postgraduate Medical Institute, Lahore, to conduct this research (23/1455). The study enrolled 120 adult patients with liver cirrhosis who required frequent LVP for managing their ascites. Participants were enrolled after providing written consent. Sample size computation was performed using OpenEpi version 3.0.0 (released 2013, developed by Andrew G. Dean, Kevin M. Sullivan, and Daniel G. Soe, Atlanta, GA, USA), with 95% confidence interval (CI) and 5% margin of error in the light of a prior study, leading to the computation of 60 patients per group [9].

Subjects aged 18 to 75 years who had known liver cirrhosis and had a recorded history of at least two LVPs in two months prior were recruited. Patients were not eligible when they had hepatocellular carcinoma, spontaneous bacterial peritonitis, decompensated cardiac or renal disease, or had taken diuretics within the last two weeks. The allocation concealment was done by assigning the patients randomly to two groups through a sealed envelope method. Group A was given 8 g of intravenous human albumin per liter of ascitic fluid drained, and Group B, an equal volume of 0.9% isotonic saline. The baseline laboratory measurements (serum creatinine and mean arterial pressure (MAP)) were noted just before every LVP session. MAP was assessed at three points after the procedure: 15 minutes, 1 hour, and 48 hours, through a standard oscillometric method. Immediate hypotensive events were defined by the lowest MAP value during the first hour after LVP.

The clinically relevant decrease in MAP 10 mmHg or more below the baseline at 48 hours was regarded as a sustained decrease. The post-paracentesis hypotension was considered when MAP <70 mmHg was maintained during the 48-hour period. The persistent hypotension was defined as the MAP measurement lower than 70 mmHg at all the post-LVP measurements. The diagnosis of PICD has adhered to the usual standard: an increase of serum creatinine 0.3 mg/dL or more above the baseline within 48 hours of LVP. The record of hospitalizations was made within 15 days after the procedure. The indications of hospitalization were categorized as hypotension-related, renal impairment, recurrent tense ascites, and other complications (e.g., infection, encephalopathy). Other endpoints were the number of patients needing repeat LVP within 15 days, the overall length of hospital stay (classified as 15 days or less, or 815 days), and symptomatic recurrence (abdominal discomfort, early satiety, or shortness of breath).

This study examined serum creatinine and MAP levels prior to and 48 hours after each session of LVP. For three months following treatment, patients were followed up. Records from clinical visits were studied to find out both the paracentesis frequency and any problems patients encountered. Data analysis was done using IBM SPSS Statistics for Windows, Version 26 (Released 2019; IBM Corp., Armonk, New York, United States). The researchers analyzed continuous variables by showing mean ± standard deviation (SD) values and independent t-tests for their comparison. Chi-square tests analyzed categorical variables. A p-value of less than 0.05 was considered statistically significant.

## Results

A sample size of 120 cirrhotic patients was equally distributed between Group A (albumin, n = 60) and Group B (saline, n = 60). The analysis of both groups revealed no statistically significant differences in mean age (54.2 ± 9.1 years vs. 53.7 ± 8.8 years) or any baseline variables, including gender distribution (39 (65.0%) vs. 37 (62.0%)), serum creatinine (1.1 ± 0.3 mg/dL vs. 1.2 ± 0.4 mg/dL), and MAP (79 ± 6.2 mmHg vs. 78 ± 7.1 mmHg) (p > 0.05). Clinical outcomes showed pronounced variations throughout the three-month study period, due to changes in paracentesis procedures and related complications. The patient group that received albumin had better health outcomes, as albumin keeps fluids stable around the kidneys during paracentesis operations. The baseline characteristics, along with their values, are given in Table 1.

**Table 1:** Baseline Characteristics of Study Participants Independent t-tests (t) were used for continuous variables, and chi-square tests (χ²) for categorical variables. A p-value < 0.05 was considered statistically significant. SD: standard deviation; n: number of participants

Variable	Group A (Albumin, n = 60)	Group B (Saline, n = 60)	Test Type	Value	df	P-value
Age (years, mean ± SD)	54.2 ± 9.1	53.7 ± 8.8	t-test	0.36	118	0.72
Male gender, n (%)	39 (65.0)	37 (62.0)	χ²	0.07	1	0.78
Serum creatinine (mg/dL)	1.1 ± 0.3	1.2 ± 0.4	t-test	1.01	118	0.32
Mean arterial pressure (mmHg)	79 ± 6.2	78 ± 7.1	t-test	0.75	118	0.45

The study groups were matched at baseline based on statistical analysis of all measured parameters. The study did not show any substantial differences between patient groups or their clinical statuses. Table 2 highlights the results of clinical outcome measures for each group at three-month follow-up.

**Table 2:** Clinical Outcomes After Three-Months Follow-Up Independent t-tests (t) were used for continuous variables, and chi-square tests (χ²) for categorical variables. A p-value < 0.001 was considered statistically significant. SD: standard deviation; n: number of participants; LVP: large-volume paracentesis; PICD: paracentesis induced circulatory dysfunction; MAP: mean arterial pressure

Variable	Group A (n = 60)	Group B (n = 60)	Test Type	Value	df	P-value
Paracentesis interval (days, mean ± SD)	18.2 ± 4.3	11.1 ± 3.7	t-test	9.85	118	<0.001
PICD, n (%)	7 (11.7)	23 (38.3)	χ²	10.86	1	0.001
↑ Serum creatinine ≥0.3 mg/dL, n (%)	6 (10.0)	18 (30.0)	χ²	7.06	1	0.008
MAP drop >5 mmHg post-LVP, n (%)	9 (15.0)	21 (35.0)	χ²	6.13	1	0.013

Patients who received albumin showed longer intervals (18.2 ± 4.3) between procedures, as well as lower rates of both PICD (7 (11.7%)) and kidney damage (6 (10.0%)), which confirmed the therapeutic benefits of albumin. Table 3 displays the rate of hospitalization and recurrence of symptoms after administration of albumin and saline in Group A and Group B.

**Table 3:** Hospitalization and Symptom Recurrence Chi-square tests (χ²) were used for categorical variables. A p-value < 0.001 was considered statistically significant. n: number of participants

Variable	Group A (n = 60)	Group B (n = 60)	Test Type	Value	df	P-value
Hospitalizations for ascites, n (%)	8 (13.3)	19 (31.7)	χ²	6.02	1	0.014
Symptom recurrence, n (%)	11 (18.3)	26 (43.3)	χ²	8.12	1	0.004
Emergency paracentesis, n (%)	5 (8.3)	17 (28.3)	χ²	9.14	1	0.002

The subjects of Group A received fewer hospital admissions (8 (13.3%)), including emergency visits. They experienced fewer recurrences of symptoms (11 (18.3%)), which demonstrated albumin's capability to stabilize patients and decrease healthcare needs. These results showed that albumin was a better therapeutic remedy for liver cirrhotic patients than saline. This highlighted the need for further research to optimize its usage in clinical settings.

## Discussion

The results of this study reinforced the emerging consensus that albumin was beneficial for the management of recurrent ascites in cirrhotic patients. Patients who received albumin required fewer sessions of paracentesis, which aligned with previous studies, in which extended paracentesis-free intervals were observed in patients treated with albumin [10]. This effect might be the result of the ability of albumin to stabilize the oncotic pressure and limit extra fluid accumulation within the peritoneal cavity, as evidenced in earlier investigations [11]. Furthermore, prior studies had also highlighted albumin’s role in promoting splanchnic vasodilation and maintaining effective plasma volume, thereby improving symptom control, reflecting and supporting the findings of this study [12].

The decrease in PICD among patients with albumin compared to those with no albumin injection agreed with many previous studies that had established the protective impact of albumin in preventing hemodynamic instability [13]. Research and clinical guidelines underscored the fact that albumin was an excellent alternative over other volume expanders in preventing PICD [14]. The current study’s findings also provided further support for these recommendations, as they indicated that lower rates of PICD in the albumin group were significantly improved, supporting the benefits of using albumin when dealing with the complications associated with LVP. This clinical benefit was also due to albumin’s unique characteristics in influencing systemic inflammation and controlling the arterial pressure [15].

Renal dysfunction, a common complication of repeated paracentesis, was particularly rare in the patients treated with albumin. This finding resonates with the earlier trials, which determined that the long-term albumin therapy reduced the rates of hepatorenal syndrome as well as prevented the kidney function from being compromised [16]. The ability of albumin to enhance renal perfusion and reduce systemic inflammation was likely underlying this protective effect. Consistency of these outcomes across multiple studies highlighted the necessity to address albumin’s dual role, both as a plasma expander and as a modulator of broader physiological processes in patients with advanced liver disease [17]. The present study also indicated that the hospitalization rates, emergency paracenteses, and symptom recurrences were significantly lower in the albumin group. Such results were in congruence with earlier studies, where utilization of albumin contributed to better stabilization of patients as well as decreased healthcare utilization [18].

Even though the study had several strengths, some limitations should also be acknowledged. The use of a single-center design confined the generalization of results across various healthcare facilities. The follow-up period was quite short, limiting an understanding of long-term outcomes like overall survival, sustained renal protection, and the health-related quality of life. Also, the economic aspect of routine albumin administration was not evaluated, despite the importance of its evaluation in terms of resource availability in economically deprived regions. It is necessary to compare multi-center peer-studies with longer follow-up periods, to include cost-benefit analyses, and evaluate whether differences exist in albumin dosing strategies.

## Conclusions

This research found that patients who received albumin showed better clinical outcomes in cirrhotic patients with recurrent ascites by extending their periods without paracentesis and decreasing complications like PICD, renal dysfunction, and readmission to hospitals. The findings justify integrating albumin into the post-paracentesis care protocols by showing that it had clear therapeutic value over saline, especially in an under-resourced setting.

Future studies should make it necessary to get comparisons with multi-center peer-studies with longer follow-up periods, to include the economic benefits, as well as to evaluate whether albumin dosing strategies are standardized or not. This could contribute to better long-term treatment and alleviate the healthcare system with advanced liver disease cases.

## References

[1] Liu YB, Chen MK (2022). Epidemiology of liver cirrhosis and associated complications: current knowledge and future directions. World J Gastroenterol.

[2] Tonon M, Piano S, Gambino CG (2021). Outcomes and mortality of grade 1 ascites and recurrent ascites in patients with cirrhosis. Clin Gastroenterol Hepatol.

[3] Tergast TL, Griemsmann M, Stockhoff L (2023). Daily low-volume paracentesis and clinical complications in patients with refractory ascites. JAMA Netw Open.

[4] Heybe MA, Mehta KJ (2024). Role of albumin infusion in cirrhosis-associated complications. Clin Exp Med.

[5] Caraceni P, O'Brien A, Gines P (2022). Long-term albumin treatment in patients with cirrhosis and ascites. J Hepatol.

[6] Sonderup MW, Kamath PS, Awuku YA (2024). Managing cirrhosis with limited resources: perspectives from sub-Saharan Africa. Lancet Gastroenterol Hepatol.

[7] Hasan I, Murti IS, Bayupurnama P, Kalista KF, Hill-Zabala C, Kananda D, Viayna E (2024). Cost-effectiveness of albumin in the treatment of decompensated cirrhosis in resource-limited healthcare settings. Drugs Context.

[8] Sandi BB, Leão GS, de Mattos AA, de Mattos ÂZ (2021). Long-term albumin administration in patients with cirrhosis and ascites: a meta-analysis of randomized controlled trials. J Gastroenterol Hepatol.

[9] Tjaden C, Mulder CL, den Hollander W (2021). Effectiveness of resource groups for improving empowerment, quality of life, and functioning of people with severe mental illness: a randomized clinical trial. JAMA Psychiatry.

[10] Yokomichi N, Imai K, Sakamoto M (2022). Feasibility of a fast-track randomized controlled trial of cell-free and concentrated ascites reinfusion therapy for patients with refractory malignant ascites. BMC Cancer.

[11] de Mattos ÂZ, Simonetto DA, Terra C (2022). Albumin administration in patients with cirrhosis: current role and novel perspectives. World J Gastroenterol.

[12] Garcia-Tsao G, Abraldes JG, Rich NE, Wong VW (2024). AGA clinical practice update on the use of vasoactive drugs and intravenous albumin in cirrhosis: expert review. Gastroenterology.

[13] Leache L, Gutiérrez-Valencia M, Saiz LC (2023). Meta-analysis: efficacy and safety of albumin in the prevention and treatment of complications in patients with cirrhosis. Aliment Pharmacol Ther.

[14] Gagliardi R, Zeni N, Piano S (2023). Intravenous albumin in cirrhosis: updated clinical uses and novel perspectives. Ann Hepatol.

[15] Bai Z, Méndez-Sánchez N, Romeiro FG (2023). Use of albumin infusion for cirrhosis-related complications: an international position statement. JHEP Rep.

[16] Jung CY, Chang JW (2023). Hepatorenal syndrome: current concepts and future perspectives. Clin Mol Hepatol.

[17] Trebicka J, Garcia-Tsao G (2025). Controversies regarding albumin therapy in cirrhosis. Hepatology.

[18] China L, Freemantle N, Forrest E (2021). A randomized trial of albumin infusions in hospitalized patients with cirrhosis. N Engl J Med.

